# To develop a machine learning-based model for predicting the risk of gastrointestinal bleeding in patients with spontaneous intracerebral hemorrhage

**DOI:** 10.3389/fneur.2025.1690638

**Published:** 2026-01-02

**Authors:** Chenzhu Cai, Jiayin Wang, Mingfa Cai, Zhen Qi, Xieli Guo

**Affiliations:** 1 Department of Neurosurgery, Jinjiang Municipal Hospital (Shanghai Sixth People’s Hospital Fujian), Jinjiang, Fujian, China; 2 Department of Neurosurgery, The Second Affiliated Hospital of Fujian Medical University, Quanzhou, Fujian, China

**Keywords:** spontaneous intracerebral hemorrhage, gastrointestinal bleeding, machine learning, intraventricular extension of hemorrhage, distance to the midline, GCS score, albumin level

## Abstract

**Background:**

Spontaneous intracerebral hemorrhage (sICH) is a critical illness with a poor clinical prognosis, and gastrointestinal bleeding (GIB) is a severe complication that can significantly worsen the patient’s adverse outcomes. However, research on the risk factors for GIB in sICH patients is currently limited. Therefore, this study aims to construct and validate a predictive model for GIB risk in sICH patients using machine learning methods, providing decision support for the early identification of high-risk patients in clinical settings.

**Methods:**

The present study retrospectively analysed the clinical data of 738 patients with sICH from two centres. In the feature selection process, the Boruta algorithm was initially employed for preliminary screening, and subsequently, the Information-Gain method was utilised to identify significant predictors. Following this, Spearman correlation analysis was implemented to eliminate collinearity between variables. During the model construction stage, the machine learning algorithm was optimized based on the internal test set, and the model performance was finally verified by the internal test set and the external validation set. In order to enhance the interpretability of the model, the SHapley Additive exPlanations (SHAP) method was used to visualize the prediction results.

**Results:**

The Glasgow Coma Scale (GCS) score, intraventricular extension of hemorrhage (ICH with IVH), surgeries, albumin, and distance to the midline were identified as significant predictors of GIB in patients with sICH. The patients were randomly divided into training and validation cohorts in an 8:2 ratio for model development and validation. An Extra Trees Classifier algorithm was used to construct the predictive model. Internal validation showed that the area under the receiver operating characteristic (ROC) curve (AUC) was 0.803 (95% CI: 0.659–0.947), while the AUC for external validation data was 0.757 (95% CI: 0.675–0.839). The calibration curves for both internal and external validation were close to the ideal diagonal line, and decision curve analysis (DCA) demonstrated that the model provided a substantial net benefit.

**Conclusion:**

Our prediction model for GIB in sICH patients has reliable predictive power and provides a reliable tool for clinicians to identify early the high-risk group for GIB in sICH patients.

## Background

Intracerebral hemorrhage (ICH) is a neurological emergency characterised by the rupture of blood vessels in the brain parenchyma, leading to haematoma formation, with a high mortality and disability rate (1, 2). According to the aetiological classification, ICH can be divided into spontaneous intracerebral hemorrhage (sICH) and secondary intracerebral hemorrhage (3). sICH refers to cerebral parenchymal hemorrhage directly caused by cerebrovascular disease (such as hypertensive arteriosclerosis or amyloid angiopathy) after excluding trauma and iatrogenic factors. Secondary intracerebral hemorrhage is caused by external factors (such as craniocerebral trauma) or systemic diseases (such as vascular malformations or tumors). In clinical practice, sICH is the most common type of intracerebral hemorrhage (3). Notably, the clinical presentation and prognosis of sICH are closely related to the location of hemorrhage. Research indicates that acute spontaneous lobar hemorrhage exhibits distinct clinical characteristics from deep subcortical hemorrhage and is typically associated with a more severe early prognosis (4). This primarily stems from differences in their pathological mechanisms: non-hypertensive mechanisms (such as cerebral amyloid angiopathy) play a dominant role in lobar hemorrhages, whereas deep hemorrhages are more frequently associated with hypertensive arteriopathy (4).

Studies have shown that the mortality rate of ICH patients within 30 days can be as high as 35 to 52% (5, 6). Common complications of ICH include pulmonary infection, gastrointestinal bleeding (GIB), central fever, epilepsy, and death. Among these, GIB is an important risk factor for a poor prognosis in ICH patients (1). The literature reports that the incidence of ICH combined with GIB is between 4.9 and 30% (7–10), and Zou et al.’s study further revealed that the mortality rate of such patients could be as high as 87.9% (11). Currently, stress ulcer (SU) is considered to be the main cause of GIB after ICH (7). SU refers to acute gastrointestinal mucosal erosion or ulcerative lesions that occur under stressful conditions, such as severe trauma, critical illness or intense mental stress. Severe SU can cause GIB and even perforation, thereby exacerbating the primary condition and significantly increasing patients’ risk of death (12, 13). For this reason, acid-suppressive drugs are often used in clinical practice to prevent stress ulcers. However, the use of SU preventive drugs is controversial because they increase medical costs and may increase the risk of nosocomial pneumonia (7). In this context, the early identification of patients at high risk of GIB after ICH and the optimization of intervention strategies are of great significance in improving patient prognosis.

This study aimed to investigate the risk of GIB in patients with sICH who did not receive SU prophylaxis. To establish a prediction model for the risk of GIB in patients with sICH, and to provide clinicians with a decision support tool for early identification of high-risk patients and timely implementation of targeted preventive measures.

## Materials and methods

### Study population

This study retrospectively collected the clinical data of 562 sICH patients admitted to the Second Affiliated Hospital of Fujian Medical University between January 2018 and December 2023 to construct a prediction model. Subsequently, the model was externally validated based on an independent dataset of 246 sICH patients admitted to Jinjiang City Hospital from January 2020 to December 2023. Inclusion criteria: (1) age > 18 years old; (2) sICH was diagnosed according to the diagnostic criteria of the Chinese College of Neurology Cerebrovascular Disease Group and confirmed by CT or MRI; (3) Admission within 24 h after symptom onset. Exclusion criteria: (1) secondary cerebral hemorrhage (such as trauma, hemorrhagic transformation after cerebral infarction, cerebrovascular malformation, brain tumor, etc.); (2) complicated with serious heart, liver, kidney and other important organ diseases; (3) previous history of GIB, hematological disease, tumor, aneurysm, cerebral arteriovenous malformation or stroke; (4) patients received surgery or interventional therapy before the onset of sICH; (5) Incomplete clinical data. After admission, each patient was examined and treated by a professional neurologist according to ICH treatment guidelines. Due to the retrospective nature of the study, ethics committee waived the need of obtaining informed consent’ in the manuscript. All methods were performed in accordance with the relevant guidelines and regulations. The study protocol was approved by the Ethics Committee of the Second Affiliated Hospital of Fujian Medical University (Ethics No.2024–677) and Jinjiang Municipal Hospital (Ethics No. jjsyyll-2025-133), and informed consent was waived.

### Data acquisition

Data on general demographic characteristics, clinical status on admission, laboratory findings, basic imaging features, and treatment details were collected from patients with sICH. Demographic data included age, sex, smoking history, and alcohol consumption. Clinical information on admission encompassed a history of hypertension and diabetes, time of onset, Glasgow Coma Scale (GCS) score, body temperature, systolic and diastolic blood pressure, respiratory rate, and heart rate. Laboratory indicators included serum albumin, white blood cell count, neutrophil count, lymphocyte count, monocyte count, haemoglobin level, platelet count, activated partial thromboplastin time (APTT), prothrombin time (PT), fibrinogen (FIB), and D-dimer levels. Imaging findings comprised haematoma volume, distance to the midline, intraventricular extension of hemorrhage (ICH with IVH), and Hematoma location (originating in the cerebral lobes as lobar, and originating in the deep brain parenchyma as deep). Treatment information included whether surgical intervention was performed. All therapeutic regimens were determined by neurosurgeons based on the patient’s specific condition and established clinical guidelines for intracerebral hemorrhage management.

### Definition of GIB

In this study, GIB was defined as any of the following conditions within 7 days after admission: (1) clinical symptoms such as hematemesis (bloody or coffee-like vomit), melena (tarry stool), or bloody stool; (2) a decrease in hemoglobin level (≥2 g/dL) from baseline accompanied by a decrease in blood pressure (systolic blood pressure <90 mmHg or a decrease in systolic blood pressure ≥20 MMHG), or laboratory evidence of vomiting, gastric drainage, or positive fecal occult blood test;(3) Active bleeding confirmed by gastroscopy, colonoscopy or imaging examinations. All diagnoses were combined with clinical manifestations and auxiliary examination results.

### Sample size calculation

To ensure the accuracy of the prediction model and avoid overfitting, this study strictly followed the internationally accepted standard for sample size calculation. According to the multivariate prediction model development criteria proposed by Riley et al., the sample size needs to meet the requirement that each candidate prediction variable corresponds to at least 10 events (EPV ≥ 10) (14). A total of 29 candidate predictors were included in this study, and at least 290 patients (29 × 10) were required according to the EPV principle. A total of 508 patients were included in the internal dataset, which were randomly divided into the training set (*n* = 356) and the validation set (*n* = 152) at a ratio of 7:3. The sample size of the training set exceeded the minimum requirement by 22.7% (356/290). The external validation cohort included 230 patients, which not only met the basic requirements of “at least 100 events in the validation set” recommended by Collins (15), but also reached the ideal validation sample size (usually recommended as 20–30% of the sample size in the development set) (16) to ensure the reliability of the model validation fully.

### Statistical analysis

Categorical variables were analysed using the Chi-square test or Fisher’s exact test, with results presented as frequency (percentage). For continuous variables that follow a normal distribution, data were described as mean ± standard deviation, and intergroup comparisons were performed using the independent samples *t*-test (after confirming normality with the Shapiro–Wilk test and homogeneity of variance with Levene’s test). Non-normally distributed variables were described as median (interquartile range), with intergroup comparisons conducted using the Mann–Whitney U test. In the data preprocessing stage, categorical variables were transformed using one-hot encoding, and SMOTE-Tomek was employed to address the issue of category imbalance. The internal dataset is randomly stratified into training and validation sets in a 8:2 ratio, and feature selection is performed in the training set queue. During feature selection, the variables with zero variance and high correlation (correlation coefficient >0.8) were removed, and then the important features were initially screened by the Boruta algorithm. The significant predictors were selected based on the Information-Gain ranking of the important features. Based on significant predictive factors, a 5-fold cross-validation was used to benchmark 17 machine learning algorithms, including Extra Trees Classifier, Random Forest Classifier, Extreme Gradient Boosting, CatBoost Classifier, and MLP Classifier. The models were evaluated using various metrics, including Accuracy, Area Under the receiver operating characteristic (ROC) Curve (AUC), Recall, Precision (Prec.), F1-Score (F1), Cohen’s Kappa (Kappa), Matthews Correlation Coefficient (MCC), Logarithmic Loss (Log Loss), Brier Score (Brier), and Training Time (Seconds) [TT(Sec)]. Through these comprehensive indicators, the best model was determined. The performance of the final model was verified by ROC curve, calibration curve and clinical decision analysis on the internal test set and independent external validation set, respectively. SHapley Additive exPlanations (SHAP) dependence map and feature importance map were used to explain the feature contributions. All statistical analyses were performed with the use of R (version 4.4.1) and Python (version 3.13.3).

## Results

### Baseline characteristics

**Figure 1:**
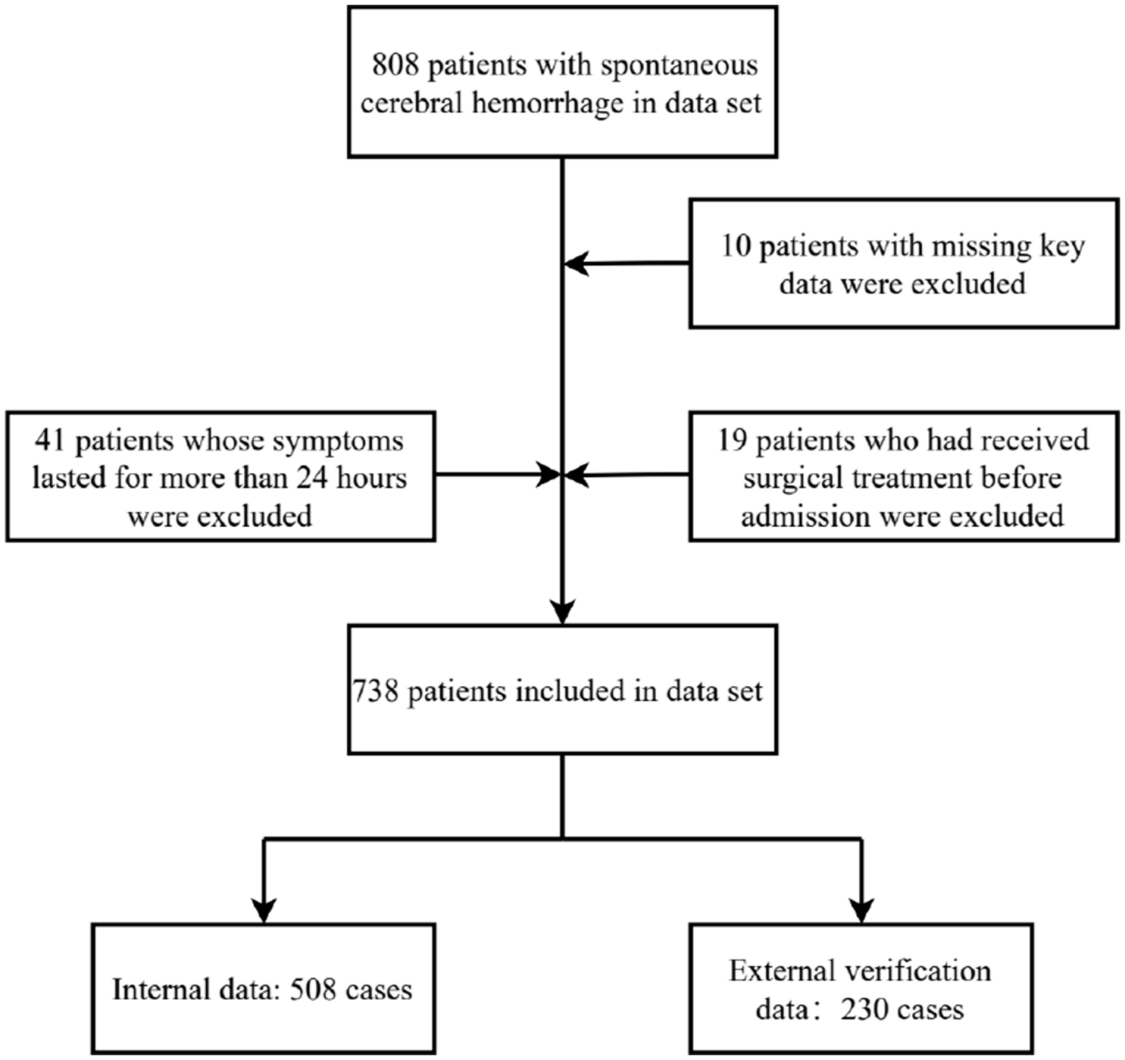
Incorporate into the patient process.

The study initially screened 808 patients who met the main diagnostic criteria. Through rigorous chart review, the following ineligible cases were excluded: 19 patients who had undergone surgery prior to admission; 41 patients whose symptoms had lasted more than 24 h; and 10 patients for whom key data were missing. A total of 738 patients were ultimately included in the study analysis, comprising 508 patients with internal data and 230 patients with external validation data (Figure 1). Table 1 presents the baseline characteristics of the internal and external validation datasets. Table 2 shows a comparison of baseline characteristics between the internal development cohort and the external validation cohort. The two groups were generally balanced across most baseline variables; however, significant differences were observed in 11 variables (*p* < 0.05), reflecting the expected heterogeneity between the patient populations.

**Table 1:** Baseline characteristics of participants.

Variable	Internal data			External data		
	Non-GIB	GIB	*p*-value	Non-GIB	GIB	*p*-value
	*N* = 419	*N* = 89		N = 185	N = 45	
Gender, n (%)			0.931			0.443
Male	261 (62.3)	55 (61.8)		134 (72.4)	30 (66.7)	
Female	158 (37.7)	34 (38.2)		51 (27.6)	15 (33.3)	
Age, Mean ± SD	56.8 ± 12.1	60.1 ± 12.4	0.022	57.5 ± 11.2	60.2 ± 10.4	0.144
Hypertension, n (%)			0.004			< 0.001
No	46 (11)	1 (1.1)		62 (33.5)	1 (2.2)	
Yes	373 (89)	88 (98.9)		123 (66.5)	44 (97.8)	
Diabetes, n (%)			0.183			0.439
No	370 (88.3)	74 (83.1)		164 (88.6)	38 (84.4)	
Yes	49 (11.7)	15 (16.9)		21 (11.4)	7 (15.6)	
Smoke, n (%)			0.066			0.568
No	274 (65.4)	49 (55.1)		163 (88.1)	41 (91.1)	
Yes	145 (34.6)	40 (44.9)		22 (11.9)	4 (8.9)	
Drinking alcohol, n (%)			0.456			1
No	344 (82.1)	76 (85.4)		167 (90.3)	41 (91.1)	
Yes	75 (17.9)	13 (14.6)		18 (9.7)	4 (8.9)	
Onset time, Median (IQR)	5.0 (3.0, 9.0)	4.0 (3.0, 6.0)	0.035	5.9 ± 3.4	5.2 ± 4.9	0.239
GCS score, Mean ± SD	9.9 ± 4.0	7.9 ± 3.1	< 0.001	10.1 ± 2.8	7.8 ± 2.8	< 0.001
Body temperature, Median (IQR)	36.6 (36.5, 36.8)	36.6 (36.5, 36.6)	0.676	36.9 ± 0.7	37.1 ± 0.9	0.162
Breathing, Mean ± SD	19.8 ± 2.4	19.2 ± 2.3	0.029	19.6 ± 3.2	18.3 ± 2.9	0.014
Heart rate, Mean ± SD	84.0 ± 15.8	80.3 ± 12.4	0.042	82.4 ± 16.2	81.2 ± 18.7	0.674
Systolic blood pressure, Mean ± SD	170.6 ± 32.1	176.5 ± 29.3	0.11	165.2 ± 19.9	177.5 ± 22.4	< 0.001
Diastolic blood pressure, Mean ± SD	95.8 ± 19.7	93.3 ± 17.4	0.269	94.4 ± 13.3	93.0 ± 17.8	0.553
Albumin, Mean ± SD	38.6 ± 6.2	35.9 ± 5.8	< 0.001	38.1 ± 4.0	36.0 ± 5.4	0.004
White blood cell, Mean ± SD	11.7 ± 4.8	12.6 ± 5.6	0.093	11.4 ± 4.0	12.1 ± 5.1	0.259
Neutrophil count, Mean ± SD	9.2 ± 4.7	10.2 ± 5.8	0.082	9.2 ± 4.0	10.0 ± 5.0	0.274
Lymphocyte count, Median (IQR)	1.4 (0.9, 2.1)	1.3 (0.9, 2.2)	0.813	3.1 ± 0.5	2.2 ± 0.8	< 0.001
Monocyte count, Median (IQR)	0.6 (0.4, 0.7)	0.6 (0.4, 0.8)	0.145	1.5 ± 0.3	1.4 ± 0.3	0.576
Hemoglobin, Mean ± SD	140.3 ± 19.3	136.2 ± 21.9	0.076	141.7 ± 23.2	136.8 ± 29.4	0.232
Platelet, Mean ± SD	238.5 ± 87.6	242.5 ± 76.5	0.695	237.5 ± 64.0	228.8 ± 62.8	0.41
PT, Mean ± SD	12.7 ± 6.2	11.4 ± 1.2	0.063	12.3 ± 3.7	11.3 ± 0.8	0.081
APTT, Mean ± SD	26.5 ± 6.5	25.3 ± 4.5	0.122	24.7 ± 5.6	26.1 ± 5.5	0.118
FIB, Median (IQR)	2.9 (2.4, 3.7)	2.6 (2.3, 3.1)	0.028	25.1 ± 5.6	25.4 ± 5.5	0.703
D-dimer, Median (IQR)	0.6 (0.3, 1.0)	0.7 (0.4, 1.0)	0.049	0.6 (0.4, 1.1)	0.6 (0.3, 1.5)	0.999
Distance to the midline, Median (IQR)	4.0 (2.0, 6.6)	6.0 (4.0, 11.0)	< 0.001	3.0 (0.0, 6.0)	7.4 (4.0, 11.0)	< 0.001
Bleeding volume, Mean ± SD	35.0 ± 21.3	43.1 ± 24.4	0.002	39.9 ± 20.0	44.8 ± 26.1	0.172
Surgeries, n (%)			< 0.001			< 0.001
No	204 (48.7)	22 (24.7)		113 (61.1)	12 (26.7)	
Yes	215 (51.3)	67 (75.3)		72 (38.9)	33 (73.3)	
ICH with IVH, *n* (%)			< 0.001			< 0.001
No	195 (46.5)	21 (23.6)		78 (42.2)	7 (15.6)	
Unilateral	138 (32.9)	60 (67.4)		72 (38.9)	32 (71.1)	
Bilateral	86 (20.5)	8 (9)		35 (18.9)	6 (13.3)	
Bleeding site, *n* (%)			0.327			0.208
Superficial	64 (15.3)	10 (11.2)		25 (13.5)	3 (6.7)	
Peripheral	355 (84.7)	79 (88.8)		160 (86.5)	42 (93.3)	

**Table 2:** Comparison of Baseline Characteristics Between the Internal Development Cohort and the External Validation Cohort.

Variable	Total data		*p*-value
	Internal data	External data	
	*N* = 508	*N* = 230	
Gender, n (%)			0.016
Male	316 (62.2)	164 (71.3)	
Female	192 (37.8)	66 (28.7)	
Age, Mean ± SD	57.4 ± 12.2	58.0 ± 11.1	0.494
Hypertension, *n* (%)			< 0.001
No	47 (9.3)	63 (27.4)	
Yes	461 (90.7)	167 (72.6)	
Diabetes, *n* (%)			0.872
No	444 (87.4)	202 (87.8)	
Yes	64 (12.6)	28 (12.2)	
Smoke, *n* (%)			< 0.001
No	323 (63.6)	204 (88.7)	
Yes	185 (36.4)	26 (11.3)	
Drinking alcohol, n (%)			0.006
No	420 (82.7)	208 (90.4)	
Yes	88 (17.3)	22 (9.6)	
Onset time, Median (IQR)	5.0 (3.0, 8.0)	6.0 (3.0, 7.0)	0.712
GCS score, Mean ± SD	9.5 ± 3.9	9.6 ± 2.9	0.733
Body temperature, Median (IQR)	36.6 (36.5, 36.7)	36.8 (36.5, 37.2)	< 0.001
Breathing, Mean ± SD	19.7 ± 2.3	19.4 ± 3.2	0.073
Heart rate, Mean ± SD	83.3 ± 15.3	82.2 ± 16.7	0.366
Systolic blood pressure, Mean ± SD	171.6 ± 31.6	167.6 ± 21.0	0.077
Diastolic blood pressure, Mean ± SD	95.4 ± 19.3	94.2 ± 14.3	0.396
Albumin, Mean ± SD	38.1 ± 6.2	37.6 ± 4.4	0.276
White blood cell, Mean ± SD	11.8 ± 5.0	11.5 ± 4.2	0.401
Neutrophil count, Mean ± SD	9.3 ± 4.9	9.4 ± 4.2	0.866
Lymphocyte count, Median (IQR)	1.7 ± 1.3	2.9 ± 0.7	< 0.001
Monocyte count, Median (IQR)	0.6 ± 0.5	1.5 ± 0.3	< 0.001
Hemoglobin, Mean ± SD	139.6 ± 19.8	140.7 ± 24.5	0.509
Platelet, Mean ± SD	239.2 ± 85.7	235.8 ± 63.7	0.588
PT, Mean ± SD	12.4 ± 5.6	12.1 ± 3.3	0.393
APTT, Mean ± SD	26.3 ± 6.2	25.0 ± 5.6	0.007
FIB, Median (IQR)	2.8 (2.3, 3.5)	24.6 (21.2, 28.8)	< 0.001
D-dimer, Median (IQR)	0.6 (0.3, 1.0)	0.6 (0.4, 1.1)	0.103
Distance to the midline, Median (IQR)	4.0 (2.0, 7.4)	4.0 (0.0, 7.0)	0.098
Bleeding volume, Mean ± SD	36.5 ± 22.1	40.9 ± 21.4	0.011
Surgeries, *n* (%)			0.013
No	226 (44.5)	125 (54.3)	
Yes	282 (55.5)	105 (45.7)	
ICH with IVH, *n* (%)			0.253
No	216 (42.5)	85 (37)	
Unilateral	198 (39)	104 (45.2)	
Bilateral	94 (18.5)	41 (17.8)	
Bleeding site, *n* (%)			0.383
Superficial	74 (14.6)	28 (12.2)	
Peripheral	434 (85.4)	202 (87.8)	

### Characteristic variable screening

**Figure 2:**
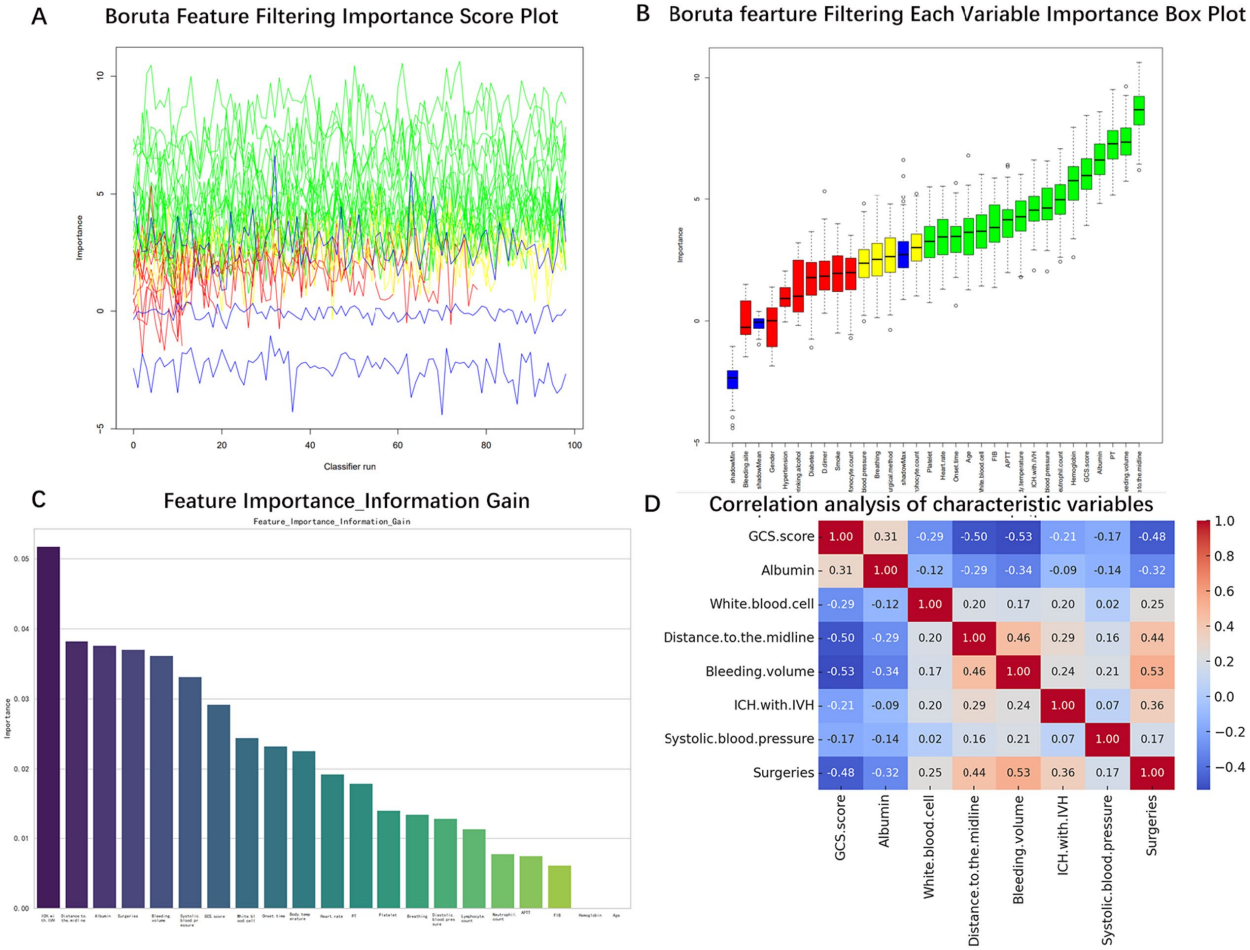
Screening process of characteristic variables. **(A)** Boruta algorithm feature selection importance score. **(B)** Boruta algorithm feature importance ranking. **(C)** Feature importance ranking of Information-Gain algorithm. **(D)** Correlation analysis between characteristic variables.

The internal dataset was randomly stratified into training and validation sets at a ratio of 8:2. In the training set, the Boruta algorithm was used to screen the features of the 29 statistical factors. By evaluating the importance of each feature, the algorithm initially screened out 21 features that significantly contributed to the occurrence of GIB in patients after sICH (Figures 2A,B). The initial screening of important features is then evaluated based on Information-Gain to assess feature importance. Firstly, the Information-Gain value between each feature and the target variable is calculated using the following formula: IG(Y, X) = H(Y) – H(Y | X), where H(Y) is the entropy of the target variable and H(Y | X) is the conditional entropy given the features. Variables with an IG threshold > 0.03 are retained (Figure 2C). Ultimately, eight variables were identified as significant predictors of GIB in patients following sICH: GCS score, albumin, white blood cell count, distance to the midline, bleeding volume, ICH with IVH, systolic blood pressure, and surgeries. Spearman correlation analysis was conducted to assess collinearity among these variables (Figure 2D). The results showed that none of the correlation coefficients exceeded 0.55, indicating no significant collinearity among the eight variables. These eight features were therefore included in the final model construction.

### Machine learning model training

**Figure 3:**
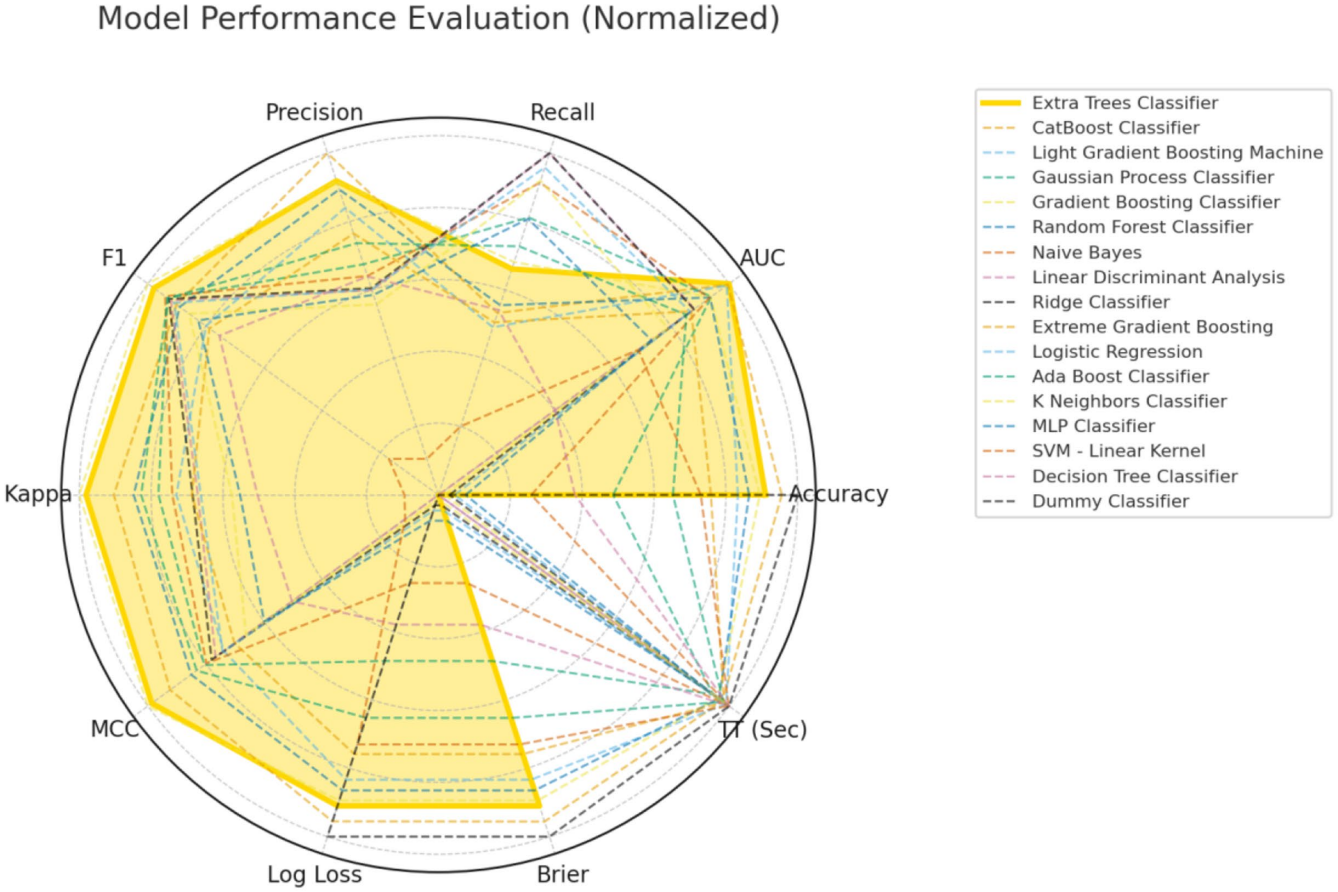
Comprehensive performance evaluation of different machine learning models.

Our model was trained based on the selected features described above. In the training set, a benchmark test was conducted on 17 machine learning algorithms, including Extra Trees Classifier, Random Forest Classifier, Extreme Gradient Boosting, CatBoost Classifier, and MLP Classifier, using 5-fold cross-validation. The models were evaluated based on Accuracy, AUC, Recall, Precision (Prec.), F1-Score (F1), Cohen’s Kappa (Kappa), Matthews Correlation Coefficient (MCC), Logarithmic Loss (Log Loss), Brier Score (Brier), and Training Time (Seconds) [TT(Sec)], as shown in Table 3. A comparison of common performance metrics for 17 machine learning prediction models was conducted. The results showed that among all the models, the Extra Trees Classifier exhibited the best predictive performance (Figure 3). Therefore, we selected the Extra Trees Classifier as the optimal model for predicting the occurrence of GIB in patients after sICH in this study.

**Table 3:** Benchmark test results.

Model	Accuracy	AUC	Recall	Prec.	F1	Kappa	MCC	Log Loss	Brier	TT (Sec)
Extra Trees Classifier	0.8105	0.7355	0.439	0.4588	0.4335	0.3232	0.3323	6.831	0.1895	3.07
CatBoost Classifier	0.8179	0.7345	0.3533	0.4992	0.399	0.2977	0.311	6.5651	0.1821	0.152
Light Gradient Boosting Machine	0.798	0.7336	0.3257	0.4194	0.3541	0.2403	0.2488	7.2793	0.202	0.112
Gaussian Process Classifier	0.7415	0.7211	0.539	0.3369	0.4129	0.2568	0.2714	9.3175	0.2585	0.248
Gradient Boosting Classifier	0.8079	0.7205	0.4524	0.4581	0.4426	0.3294	0.3368	6.9233	0.1921	0.196
Random Forest Classifier	0.8031	0.7202	0.3686	0.4463	0.3941	0.2795	0.2866	7.0959	0.1969	0.186
Naive Bayes	0.7044	0.7196	0.6067	0.3198	0.4163	0.2441	0.2688	10.6557	0.2956	0.082
Linear Discriminant Analysis	0.6624	0.7074	0.6638	0.2991	0.4075	0.2206	0.2583	12.1676	0.3376	0.084
Ridge Classifier	0.6674	0.707	0.6638	0.302	0.4106	0.2254	0.2629	11.9896	0.3326	0.082
Extreme Gradient Boosting	0.7858	0.7048	0.3552	0.3818	0.3489	0.2262	0.2364	7.7199	0.2142	0.136
Logistic Regression	0.6698	0.7041	0.6362	0.3003	0.4025	0.2179	0.2502	11.9006	0.3302	0.124
Ada Boost Classifier	0.7686	0.7008	0.4829	0.3678	0.4121	0.2726	0.2802	8.3418	0.2314	0.142
K Neighbors Classifier	0.6651	0.6859	0.6105	0.2783	0.3799	0.1894	0.2232	12.071	0.3349	0.088
MLP Classifier	0.6747	0.6726	0.5362	0.2923	0.3657	0.1819	0.202	11.7248	0.3253	0.284
SVM—Linear Kernel	0.7812	0.6619	0.1333	0.0526	0.0755	0.0312	0.0386	7.8849	0.2188	0.086
Decision Tree Classifier	0.7242	0.5946	0.3952	0.2917	0.3337	0.166	0.1697	9.9416	0.2758	0.086
Dummy Classifier	0.8251	0.5	0.0	0.0	0.0	0.0	0.0	6.3025	0.1749	0.082

### Model testing

**Figure 4:**
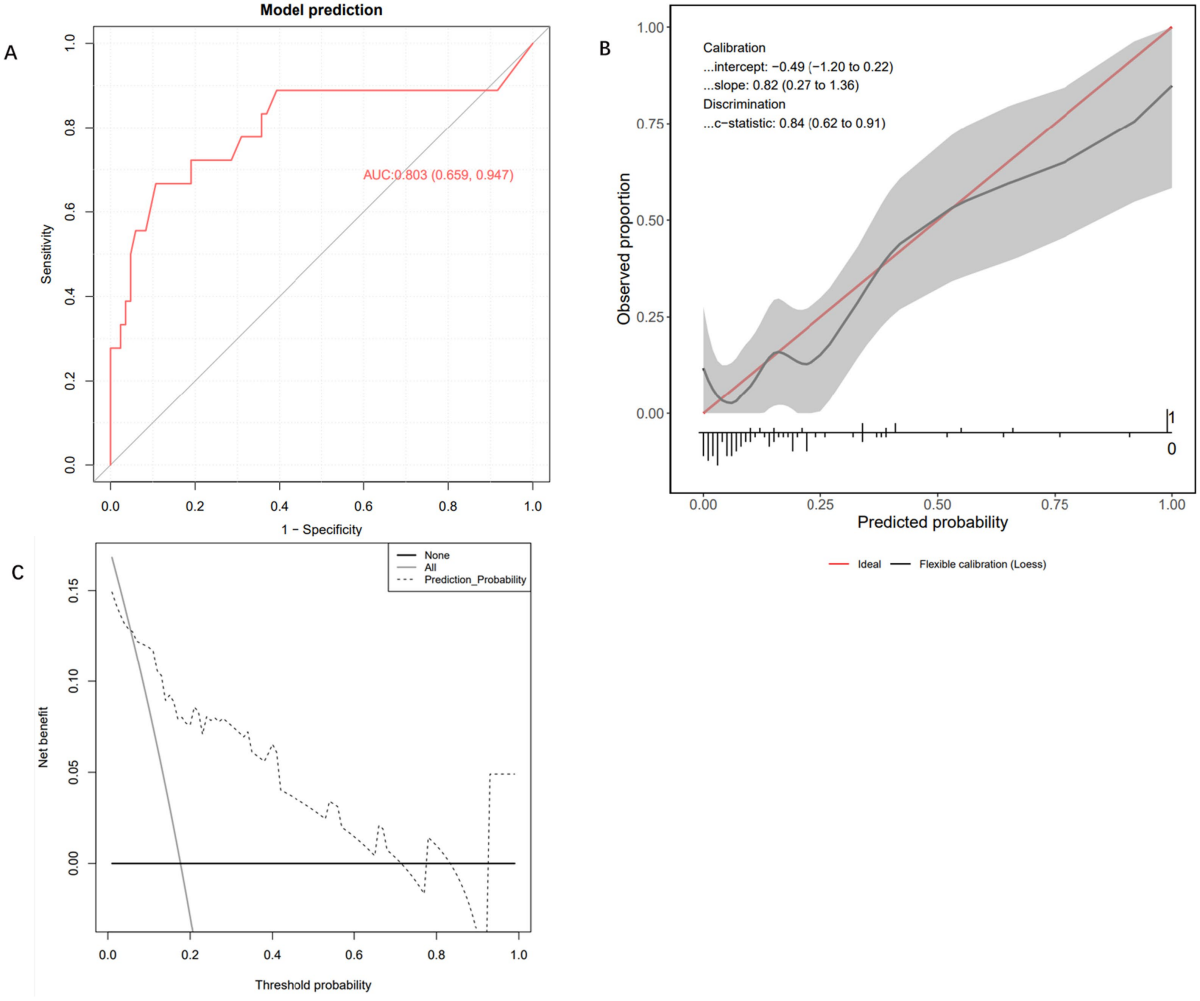
Internal testing and performance evaluation of the model. **(A)** Receiver operating characteristic curve of the Extra Trees Classifier machine learning model. **(B)** Calibration curve of the Extra Trees Classifier machine learning model. **(C)** Decision curve analysis curve of the Extra Trees Classifier machine learning model.

**Figure 5:**
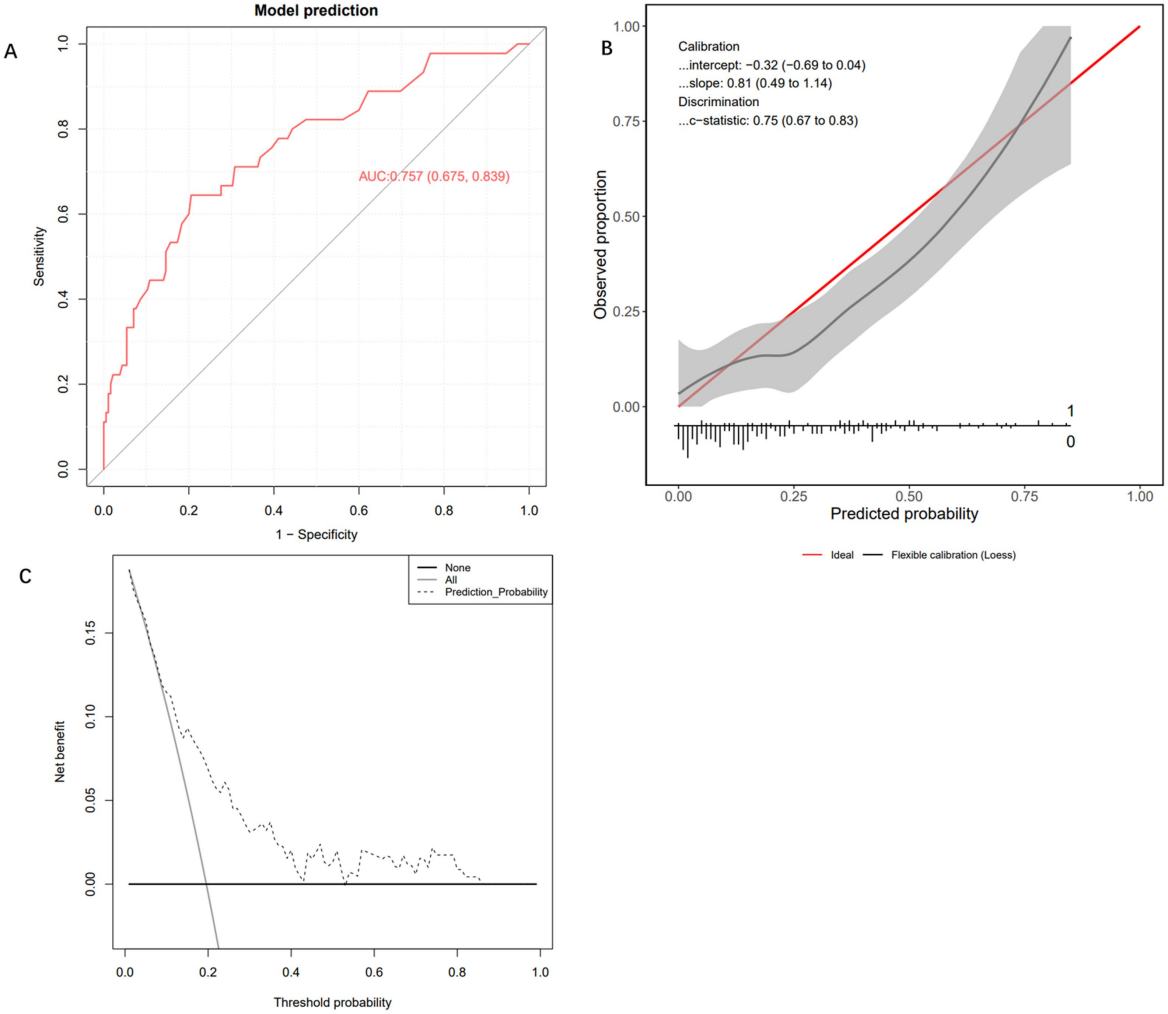
External testing and performance evaluation of the model. **(A)** Receiver operating characteristic curve of the Extra Trees Classifier machine learning model. **(B)** Calibration curve of the Extra Trees Classifier machine learning model. **(C)** Decision curve analysis curve of the Extra Trees Classifier machine learning model.

To evaluate the performance of the Extra Trees Classifier model, we conducted comprehensive testing using both an internal test set and an external validation set. This assessment covered the model’s discriminative ability, calibration, and net benefit analysis across different probability thresholds. Evaluation on the internal test set showed that the AUC of the ROC curve was 0.803 (95% CI: 0.659, 0.947), indicating excellent performance in distinguishing between positive and negative classes (Figure 4A). The calibration curve demonstrated that the model’s predictions closely followed the ideal diagonal line (Figure 4B), with a c-statistic of 0.84 (95% CI: 0.62, 0.91), further confirming good calibration performance. In addition, decision curve analysis (DCA) revealed that the model’s performance was close to the “Oracle” strategy — the strategy that maximizes net benefit—suggesting that the classifier can effectively support clinical decision-making (Figure 4C). Subsequently, we further evaluated the model’s performance using the external validation cohort. In the independent external validation, despite differences in patients’ baseline characteristics, the model maintained good predictive performance, with an AUC of 0.757 (95% CI: 0.675–0.839) (Figure 5A). The calibration curve demonstrated a high degree of agreement between the predicted risk and the observed outcomes, indicating good calibration, with a C-statistic of 0.75 (95% CI: 0.67–0.83), confirming the model’s stable calibration ability in the external validation cohort (Figure 5B). Moreover, decision curve analysis revealed that the model’s performance closely approximated the “Oracle” strategy (red dashed line), suggesting that the model could provide valuable support for clinical decision-making (Figure 5C). Taken together, these findings indicate that the Extra Trees Classifier model possesses considerable clinical potential for predicting GIB in patients following sICH.

### Illustration of the extra trees classifier model using the SHAP method

**Figure 6:**
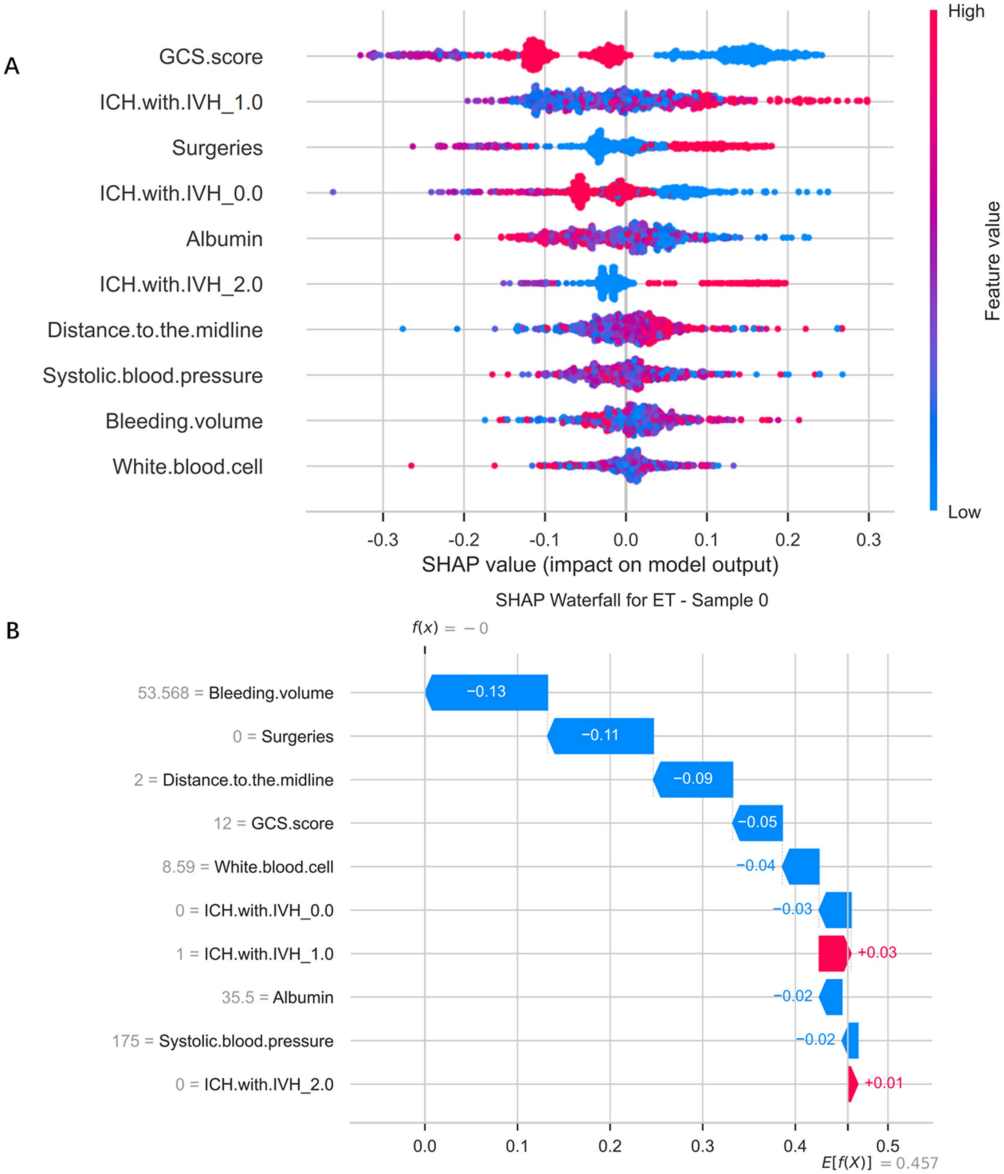
SHapley Additive exPlanations analysis of the Extra Trees Classifier model predicting gastrointestinal bleeding in patients after spontaneous intracerebral hemorrhage. **(A)** SHapley Additive exPlanations distribution plot of Extra Trees classifier model. **(B)** Significance map of a single sample feature for a selected patient.

Figure 6A illustrates the distribution of SHapley Additive exPlanations (SHAP) values based on the Extra Trees Classifier, which was used to interpret both the direction and magnitude of each feature’s contribution to the predictive model. It can be observed that different features exert markedly varying influences on the model’s output. Specifically, different categories of ICH with IVH exhibited a clear risk gradient: when no intraventricular hemorrhage was present (IVH_0.0), SHAP values were predominantly negative, suggesting a protective effect; however, when unilateral (IVH_1.0) or bilateral (IVH_2.0) intraventricular hemorrhage occurred, the SHAP values gradually shifted to positive, indicating that the presence of intraventricular bleeding substantially increased the predicted risk. Higher GCS scores (red regions in the plot) corresponded to negative SHAP values, while lower scores (blue regions) corresponded to positive SHAP values, demonstrating that poorer neurological status was associated with higher predicted risk. Surgical treatment (red dots) was associated with positive SHAP values, suggesting it as one of the risk factors. Albumin levels displayed a clear negative correlation: lower levels (blue) significantly increased SHAP values, indicating that hypoalbuminaemia markedly elevated predicted risk. The distance to the midline also showed a positive association — greater displacement (red dots) corresponded to higher positive SHAP values, implying that more pronounced brain shift was linked to increased risk. Additionally, systolic blood pressure, bleeding volume, and white blood cell count contributed to the model’s predictions, though their overall influence was comparatively smaller. To further enhance visual interpretation, Figure 6B presents a SHAP bar chart generated from a randomly selected patient sample, used to explain the model’s prediction process and the impact of each feature on that specific case. The baseline value of 0.00 represents the model’s predicted value in the absence of any feature influence. The direction and length of each bar reflect the feature’s contribution — bars extending to the right (red) indicate a positive impact, whereas those to the left (blue) indicate a negative impact. Specifically, the SHAP values for the features Bleeding volume (53.568), Surgeries (0), Distance to the midline (2 mm), GCS score (12), White blood cell (8.59), ICH with IVH_0.0 (0), ICH with IVH_1.0 (1), Albumin (35.5), Systolic blood pressure (175), and ICH with IVH_2.0 (0) were −0.13, −0.11, −0.09, −0.05, −0.04, −0.03, 0.03, −0.02, −0.02, and 0.01, respectively, indicating varying degrees of contribution to the prediction outcome. This figure provides an intuitive depiction of how the model derives its predictions from individual features, thereby enhancing the interpretability of the model.

## Discussion

GIB is a significant complication that severely impacts the prognosis of patients after ICH. Studies have shown that the incidence of GIB following ICH ranges from 4.9 to 30% (6–10). In this study, among 738 sICH patients who did not receive SU prophylaxis, the GIB incidence was 18.16% (134/738), a result that is generally consistent with the overall incidence of GIB following ICH reported in the literature. As none of the enrolled patients used SU prophylactic medications, the data from this study provide an accurate reflection of the natural incidence of GIB in sICH patients. If timely and effective preventive or therapeutic measures are not implemented, GIB following sICH can lead to rapid deterioration of the patient’s condition, increasing treatment difficulty and the risk of death (17, 18). Investigating the risk factors for GIB in sICH patients and establishing a predictive model is of significant importance.

This study is the first to develop a predictive model for assessing the risk of GIB in patients with sICH. The research team retrospectively analysed clinical data from 738 patients with sICH, including 134 who developed GIB and 604 who did not. The results showed that unilateral or bilateral ICH with IVH, surgical treatment, and distance to the midline were positively associated with the risk of GIB, whereas GCS score, absence of ICH with IVH, and albumin level were negatively associated with the risk. Systolic blood pressure, bleeding volume, and white blood cell count contributed relatively less to the prediction outcome. Internal validation demonstrated that the model achieved an area under the ROC curve (AUC of 0.803), with calibration curves closely aligning with the ideal diagonal, and DCA indicating a significant net benefit. Notably, the model maintained good predictive performance (AUC 0.757) when tested on an independent cohort that differed substantially in baseline characteristics from the development cohort. Rather than diminishing the model’s value, this finding confirmed that it had successfully learned generalisable predictive patterns from the training data and demonstrated robustness in handling real-world patient heterogeneity. This model provides a reliable tool for the early clinical identification of patients with sICH who are at high risk of developing GIB.

This study found that the GCS score is a significant risk factor for GIB in patients with sICH, a finding consistent with the results reported by Chen et al. (18) and Yang et al. (7). The GCS score can be regarded as a comprehensive indicator reflecting both neurological function and systemic stress status, with its underlying mechanism primarily attributed to systemic physiological disturbances triggered by severe brain injury. Firstly, a low GCS score, indicative of severe neurological impairment, directly disrupts central autonomic regulation, leading to excessive activation of the hypothalamic–pituitary–adrenal (HPA) axis and heightened sympathetic nervous activity, which in turn increases catecholamine secretion (19). This disturbance of the “brain–gut axis” causes marked vasoconstriction of the gastrointestinal mucosal vessels, resulting in local ischaemia, hypoxia, and redistribution of blood flow. Within hours, these processes can give rise to stress-related mucosal ulcers and erosions, establishing the pathological foundation for GIB (20). Secondly, a low GCS score is often accompanied by impaired consciousness and dysphagia, necessitating invasive procedures such as nasogastric feeding, which may mechanically damage the gastrointestinal mucosal barrier. In addition, a low GCS score is typically associated with elevated intracranial pressure, more extensive brain injury, and a stronger systemic inflammatory response syndrome. The activation of systemic inflammation further aggravates mucosal hypoperfusion and oxidative stress, while inflammatory mediators such as tumour necrosis factor-*α* (TNF-α) and interleukin-6 (IL-6) directly injure mucosal cells and inhibit their repair capacity, creating a vicious cycle (21, 22). Therefore, as a comprehensive measure of neurological function, a low GCS score reflects the combined effect of multiple pathogenic mechanisms. This explains its consistent role as a significant predictive factor in both our model and previous studies.

In this study, the machine learning model identified two key imaging predictors—ICH with IVH and distance to the midline—both of which are pathophysiologically interconnected and jointly contribute to an increased risk of GIB following sICH. Firstly, studies have shown that intraventricular extension of a parenchymal haematoma is a major determinant of poor prognosis, as it obstructs cerebrospinal fluid pathways, leading to acute obstructive hydrocephalus and a rapid rise in intracranial pressure (23). This abrupt increase in intracranial pressure strongly stimulates the central nervous system, triggering excessive sympathetic activation and a surge in catecholamine release, which in turn induces vasoconstriction and acute ischaemia of the gastrointestinal mucosa (24, 25). Secondly, the degree of distance to the midline directly reflects the severity of the mass effect within one cerebral hemisphere and serves as a precursor to raised intracranial pressure and brain herniation. A pronounced distance to the midline not only indicates severe primary brain injury but also suggests global cerebral hypoperfusion and brainstem compression, which can further deepen consciousness impairment (i.e., lower GCS scores) and severely disrupt the stability of the brain–gut axis (1). Moreover, these pathological processes (IVH and distance to the midline) may collectively exacerbate systemic inflammatory responses and alter gut microbiota homeostasis. Evidence suggests that excessive sympathetic activation and dysfunction of the brain–gut axis following sICH can lead to gut dysbiosis, characterized by the proliferation of harmful bacterial species and compromised intestinal barrier integrity (26, 27). These alterations further activate systemic inflammatory responses, releasing large amounts of inflammatory mediators that, in turn, aggravate local mucosal injury in the gastrointestinal tract, creating a vicious cycle and markedly increasing the risk of GIB (26, 27). In summary, ICH with IVH and distance to the midline may synergistically elevate the risk of GIB through multiple interrelated mechanisms, including increased intracranial pressure, heightened neuroendocrine activation, disruption of the brain–gut axis, and secondary gut microbiota–immune dysregulation.

Serum albumin is an important indicator of a patient’s nutritional status, particularly in cases of acute or critical illness. Low levels of albumin are commonly associated with poor nutritional status (28). In our study, we found that patients with decreased albumin levels were more prone to GIB. Low albumin levels may indicate underlying conditions such as malnutrition or liver dysfunction, which can make gastrointestinal blood vessels more fragile and increase the risk of bleeding. Albumin plays a crucial role in maintaining plasma osmotic pressure (29, 30). A decrease in its levels leads to a reduction in plasma osmotic pressure, which can result in edema, fluid accumulation in tissues, and increased vascular permeability. These physiological changes may disrupt the microcirculation of the gastrointestinal tract, causing mucosal damage and increasing the tendency for bleeding. In addition, serum albumin levels during the acute phase can reflect the patient’s inflammatory status (31–34). A decrease in albumin levels may be associated with a systemic inflammatory response, which can further damage the vascular walls and increase vascular permeability. This can facilitate GIB. Therefore, a reduction in albumin levels signifies not only malnutrition, but also increases the risk of GIB due to its impact on plasma osmotic pressure, vascular permeability and inflammation.

This study also found that surgical intervention is an independent risk factor for GIB following sICH. This may be because surgery itself acts as a physiological stressor, exacerbating the existing sympathetic overactivity and inflammatory response that occur after sICH, thereby further damaging the gastrointestinal mucosal barrier. In addition, fluctuations in coagulation function during the perioperative period, the use of anticoagulant medications, and postoperative fasting collectively contribute to a markedly increased risk of GIB. Therefore, for surgical patients—regardless of their baseline preoperative risk—the perioperative period should be regarded as a critical window for GIB prevention, during which appropriate prophylactic measures should be implemented.

In this study, unilateral or bilateral ICH with IVH, surgical intervention, and distance to the midline were positively associated with the risk of GIB, whereas GCS score, absence of ICH with IVH, and albumin level were negatively associated with the risk. However, the intrinsic relationships and synergistic effects among these factors also warrant in-depth exploration. Unilateral or bilateral ICH with IVH not only directly exacerbates brain tissue injury and elevates intracranial pressure but may also activate systemic stress responses and the sympathetic nervous system, leading to reduced gastrointestinal mucosal blood flow and impairment of the mucosal barrier, thereby increasing the risk of GIB. When this pathological process is accompanied by surgical intervention, the trauma of surgery, anaesthetic stress, and postoperative inflammatory responses may further amplify mucosal vulnerability, producing a cumulative effect. The degree of distance to the midline, as an important indicator of raised intracranial pressure and the severity of brain injury, often reflects more pronounced cerebral oedema and neurological deterioration. These changes may indirectly trigger or aggravate gastrointestinal mucosal ischaemia, erosion, and even bleeding through neuroendocrine activation and the release of inflammatory mediators. Meanwhile, a reduced GCS score indicates deeper levels of consciousness disturbance and more severe neurological impairment. Such patients often exhibit autonomic dysfunction and systemic metabolic imbalance, which not only heighten susceptibility to stress-related ulcers but also compromise gastrointestinal defence mechanisms, thereby substantially increasing the risk of GIB. Conversely, the absence of ICH with IVH may offer a degree of protection for gastrointestinal mucosal integrity and functional stability, as it is typically associated with milder intracranial pathology and a lower systemic inflammatory burden. Albumin level, as a key indicator reflecting nutritional status, hepatic synthetic function, and systemic inflammation, contributes to maintaining intravascular oncotic pressure, reducing tissue oedema, and exerting anti-inflammatory and antioxidant effects when within the normal or higher range — all of which enhance mucosal repair and defence. In contrast, hypoalbuminaemia is often linked to capillary leakage, delayed mucosal healing, and immunosuppression, which collectively promote the onset and progression of GIB. Importantly, these factors do not exist in isolation but rather interact in a complex pathophysiological network. For example, surgical intervention and greater distance to the midline may jointly contribute to further reductions in GCS score, while low albumin levels may form a vicious cycle with severe ICH with IVH and surgical stress, continually heightening GIB risk. Therefore, in the clinical management of patients with sICH, special attention should be paid to those presenting with ICH with IVH, requiring surgical intervention, exhibiting marked distance to the midline, reduced GCS scores, or declining albumin levels. Comprehensive preventive strategies—including the use of stress ulcer prophylaxis—should be implemented to effectively reduce the risk of GIB. However, there are several limitations to our study. Firstly, the study used a retrospective approach to analyse data, relying on patients’ past records and clinical data. This design may introduce selection and information biases, particularly with regard to data collection and clinical assessments, which may have overlooked potential confounding factors. Secondly, while this study reveals correlations between different factors and the occurrence of GIB, the study design limits our ability to definitively establish causal relationships between these factors. Future prospective studies and randomized controlled trials may be more helpful in verifying these causal relationships.

## Conclusion

This study developed a machine learning model to predict the risk of GIB in sICH patients and found that unilateral or bilateral ICH with IVH, surgical intervention, midline shift distance, and decreased albumin levels were positively correlated with the risk of GIB. Therefore, timely monitoring and intervention of these key clinical parameters may help in the early identification of high-risk patients, enabling the development of personalized prevention strategies to reduce GIB occurrence and improve patient outcomes. Although this study provides valuable insights into the prediction of GIB risk in sICH patients, further validation of these findings is needed through larger-scale prospective studies. Additionally, exploring more potential risk factors will be essential to improve the clinical management of sICH.

## Data Availability

The datasets presented in this article are not readily available because the data contains patient information and must be kept confidential. Requests to access the datasets should be directed to Chenzhu Cai, 13959852928@163.com.
